# The Role of High-Mobility Group Box 1/Toll-Like Receptor 4 in Pericardial Fibrosis and Postoperative Low Cardiac Output Syndrome in Constrictive Pericarditis

**DOI:** 10.14740/cr2228

**Published:** 2026-06-05

**Authors:** Li Kui Fang, Tian Xiang Wang, Fang Ming Zhong, Guo Can Yu, Wei Hua Li

**Affiliations:** a Department of Thoracic Surgery, Hangzhou Red Cross Hospital, Hangzhou 310003, China; b Department of Pathology, Hangzhou Red Cross Hospital, Hangzhou 310003, China

**Keywords:** Constrictive pericarditis, Low cardiac output syndrome, TLR4, HMGB1

## Abstract

**Background:**

It has remained unclear about the factors that are involved in the pericardial fibrotic process and the occurrence of postoperative low cardiac output syndrome (LCOS) in constrictive pericarditis. This study aimed to analyze the role of toll-like receptor 4 (TLR4) and high-mobility group box 1 (HMGB1) in pericardial fibrosis, and their impact on the development of postoperative LCOS in patients with constrictive pericarditis.

**Methods:**

This retrospective study enrolled 24 constrictive pericarditis patients who underwent isolated pericardiectomy at our department from May 2023 to April 2025. Pericardial tissues were subjected to immunohistochemistry to detect the expression of TLR4, HMGB1, α-smooth muscle actin (α-SMA) and collagen III. Mean optical density (MOD) was used for quantitative analysis of immunohistochemical staining. Pearson correlation analysis was performed to assess associations between TLR4, HMGB1 and fibrotic markers, while receiver operating characteristic (ROC) curves were used to evaluate their potential predictive value for postoperative LCOS.

**Results:**

Of the 24 patients, seven (29.2%) patients developed postoperative LCOS. TLR4 was expressed in 21 (87.5%) specimens, and HMGB1 was expressed in all specimens. Pearson correlation analysis showed positive correlations between TLR4 and α-SMA (r = 0.529, P = 0.008), HMGB1 and α-SMA (r = 0.516, P = 0.010), and TLR4 and HMGB1 (r = 0.844, P < 0.001). MOD values of TLR4 and HMGB1 were significantly higher in patients with postoperative LCOS (P = 0.028 and P < 0.001, respectively). ROC curve suggested that TLR4 (area under the curve (AUC) = 0.790, 95% confidence interval (CI), 0.516–1.000) and HMGB1 (AUC = 0.941, 95% CI, 0.853–1.000) had potential predictive value for postoperative LCOS.

**Conclusions:**

TLR4 and HMGB1 were involved in the pericardial fibrosis and were significantly associated with the occurrence of postoperative LCOS in constrictive pericarditis.

## Introduction

Constrictive pericarditis is a rare but life-threatening disease, and the most common cause is tuberculosis worldwide [1]. Fibrosis is the core pathological feature of constrictive pericarditis, which ultimately results in diastolic heart failure [2]. Pericardiectomy is the only definitive treatment option but is accompanied with high perioperative risk [3, 4]. Low cardiac output syndrome (LCOS) is the main postoperative complication and a major cause of postoperative mortality [5–7]. At present, it has remained unclear about the factors that are involved in the pericardial fibrotic process and the occurrence of postoperative LCOS.

Toll-like receptors (TLRs) are a class of transmembrane structural proteins that play a crucial role in the innate immune system [8]. Studies have shown that they may participate in the activation of fibroblasts, and among them, TLR4 has been proven to exert a significant pro-fibrotic effect [9–11]. In rat constrictive pericarditis model, activation of the TLR4 signaling pathway stimulates the expression of α-smooth muscle actin (α-SMA) and promotes the process of myocardial fibrosis [12]. High-mobility group box 1 (HMGB1) is a highly conserved nuclear protein that is widely expressed in nearly all cell types, and a growing body of research has shown that HMGB1 is closely associated with various fibrotic diseases [13, 14]. TLR4 is one of the most extensively studied receptors for HMGB1, and inhibiting the expression of HMGB1 could inactivate the HMGB1/TLR4 pathway to impede the development of fibrosis [15].

However, the expression patterns and functional roles of TLR4 and HMGB1 in the pericardial tissue of patients with constrictive pericarditis have remained unclear. We aimed to analyze the expression of TLR4 and HMGB1 in pericardial tissue, the associations between their expression and that of key fibrotic proteins, α-SMA and collagen III (Col-III), as well as their impact on the development of postoperative LCOS in patients with constrictive pericarditis.

## Materials and Methods

### Study population

A retrospective analysis was conducted on the clinical records of patients diagnosed with constrictive pericarditis who were treated in our department between May 2023 and April 2025. Pericardial tissues surgically resected from 24 eligible patients were selected for immunohistochemical staining. No patient had histories of autoimmune or established connective tissue diseases.

### Ethics approval and consent to participate

The study protocol was approved by the Institutional Review Board of Hangzhou Red Cross Hospital (No. 2025149-001). Because of the retrospective nature of the study and the absence of any specific intervention, the requirement for informed consent was waived. All data were maintained with confidentiality. The present study complied with the Declaration of Helsinki.

### Surgical procedure

Main preoperative therapeutic regimens of the study patients were shown here (Supplementary Material 1, cr.elmerpub.com). All the patients underwent general anesthesia, followed by pericardiectomy conducted via median sternotomy with no cardiopulmonary bypass employed during the procedure. The surgical resection extent covered at least the anterolateral pericardium located between the two phrenic nerves, the basal pericardium on the diaphragmatic surface, the pericardium on the great arteries, and the pericardium extending from the superior vena cava-right atrium junction to the inferior vena cava-right atrium junction.

### Postoperative outcomes

Postoperative outcomes of all enrolled patients were documented, with postoperative LCOS designated as the primary outcome event. LCOS presented with myocardial dysfunction alongside a cardiac index < 2.0 L/min/m^2^, systolic blood pressure < 90 mm Hg, and confirmed tissue hypoperfusion in patients without hypovolemia [16]. The postoperative time window used for LCOS diagnosis was limited to the ICU stay.

### Immunohistochemistry

Pericardial tissue sections were stained with hematoxylin and eosin (H&E). The paraffin-embedded tissue sections were firstly deparaffinized, followed by antigen retrieval and blocking. The sections were incubated with primary antibody against α-SMA (dilution ratio: 1:1,500; #14395-1-AP, ProteinTech), Col-III (dilution ratio: 1:500; #22734-1-AP, ProteinTech), TLR4 (dilution ratio: 1:400; #19811-1-AP, ProteinTech) and HMGB1 (dilution ratio: 1:200; #10829-1-AP, ProteinTech), followed by incubation with a secondary antibody, color development and counterstaining. The procedures were conducted via the Roche Automated Multifunctional Histopathological Detection System (BenchMark XT). The stained sections were examined by two pathologists, who were blinded to postoperative outcomes. All tissue sections after immunohistochemical staining were subjected to image acquisition under the same microscope and camera settings. The two pathologists jointly reviewed the stained sections and reached a consensus to randomly select three non-overlapping, representative fields of view for each sample.

### Statistical analysis

The mean optical density (MOD) was used for quantitative analysis of the immunohistochemically stained images. Image-pro Plus 6.0 software (Media Cybernetics, Inc., Rockville, MD, USA) was employed, with the same brown-yellow color set as the uniform criterion for determining positivity in all images. For each image, the integrated optical density (IOD) of positive areas and the pixel area (AREA) of the tissue were measured. The MOD value was then calculated using the formula: MOD = IOD/AREA. Categorical data were compared using the Chi-square test, the corrected Chi-square test or the Fisher exact test, and continuous data using the *t*-test or Mann–Whitney U test depending on the actual condition. Pearson correlation analysis was performed to evaluate the correlations between TLR4, HMGB1 and key fibrosis-related proteins, respectively. Receiver operating characteristic (ROC) curve analysis and the area under the curve (AUC) were used to assess the potential diagnostic value of TLR4 and HMGB1 for postoperative LCOS. Statistical analyses were conducted using SPSS 27.0 software (IBM SPSS Inc., Chicago, IL, USA) and R 4.4.0 software (The R Project for Statistical Computing). All statistical tests were two-tailed, and a P value < 0.05 was considered statistically significant.

## Results

### Clinical characteristics

A total of 24 patients receiving isolated pericardiectomy were included in this study, and seven (29.2%) cases had postoperative LCOS. The clinical characteristics were presented in Table 1. Of the 24 patients, 22 (91.7%) had tuberculosis as the etiological factor, while the remaining two (8.3%) patients were considered to have idiopathic constrictive pericarditis.

**Table 1:** Clinical Characteristics of the Study Population

Variables	N = 24
Age, years	65.5 (18.0–83.0)
Sex	
Male	18 (75.0%)
Female	6 (25.0%)
Etiology	
Tuberculosis	22 (91.7%)
Idiopathic	2 (8.3%)
NYHA functional class	
I	0 (0%)
II	10 (41.7%)
III	11 (45.8%)
IV	3 (12.5%)
BMI, kg/m^2^	21.7 (15.6–31.5)
CVP, cm H_2_O	24.0 (16.0–36.4)
Pericardial thickness, mm	8.6 (3.5–18.4)
LVEF, %	58.3 (42.0–68.6)
Postoperative LCOS	7 (29.2%)

Values presented as n (percentage) for categorical variables and median (range) for continuous variables. NYHA: New York Heart Association; BMI: body mass index; CVP: central venous pressure; LVEF: left ventricular ejection fraction (measured on echocardiogram); LCOS: low cardiac output syndrome.

### Pathological and immunohistochemical features

**Figure 1:**
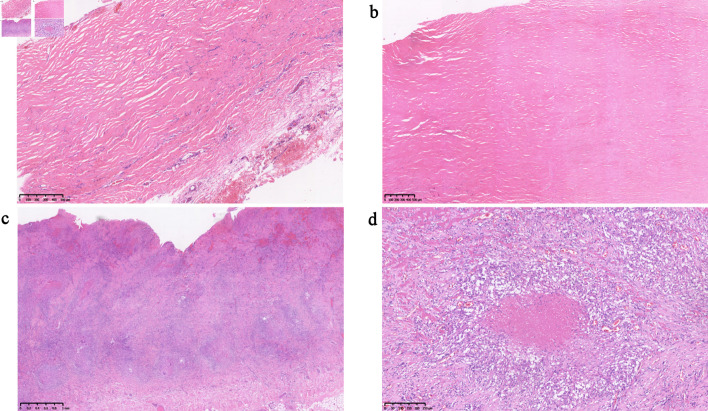
Hematoxylin and eosin (H&E) stained sections of patients with chronic constrictive pericarditis. (a, b) Microscopic findings of pericardial tissue sections from patients with nonspecific pericarditis (scale bars = 500 µm for both). (c, d) Microscopic findings of pericardial tissue sections from patients with tuberculous pericarditis (scale bars = 1 mm and 250 µm, respectively).

All patients exhibited fibrotic changes in their pericardial tissue. Among them, abundant collagen fibrous tissue was observed in the tissue sections of patients with idiopathic constrictive pericarditis, whereas massive inflammatory cell infiltration, granulomatous components and coagulative necrosis were noted in those of patients with tuberculous constrictive pericarditis (Fig. 1).

**Figure 2:**
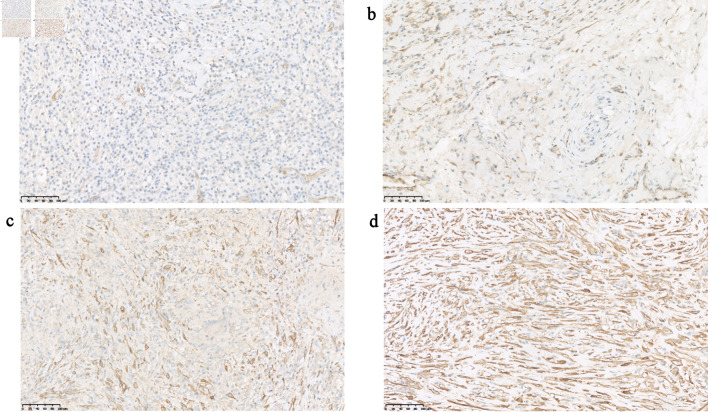
Expression of α-SMA in pericardial tissue from different patients (scale bars = 100 µm for all panels). α-SMA: α-smooth muscle actin.

**Figure 3:**
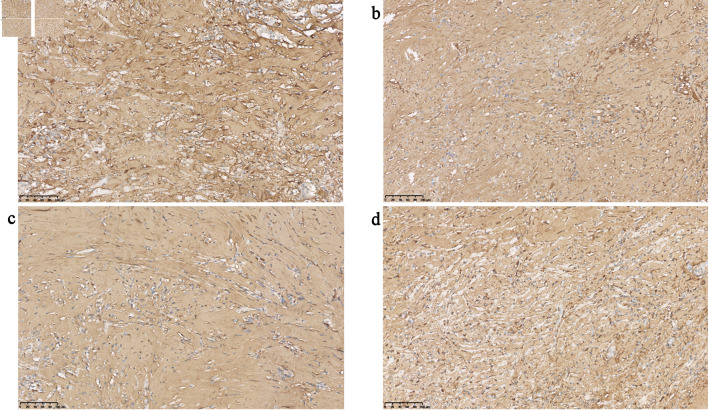
Expression of Col-III in pericardial tissue from different patients (scale bars = 100 µm for all panels). Col-III: collagen III.

Immunohistochemical staining showed that α-SMA and Col-III were expressed in the pericardial tissue of all patients. Among these patients, significant differences were observed in the expression levels of α-SMA in pericardial tissue (Fig. 2). In contrast, Col-III was abundantly expressed in the pericardial tissue of all patients, with no significant differences noted (Fig. 3).

**Figure 4:**
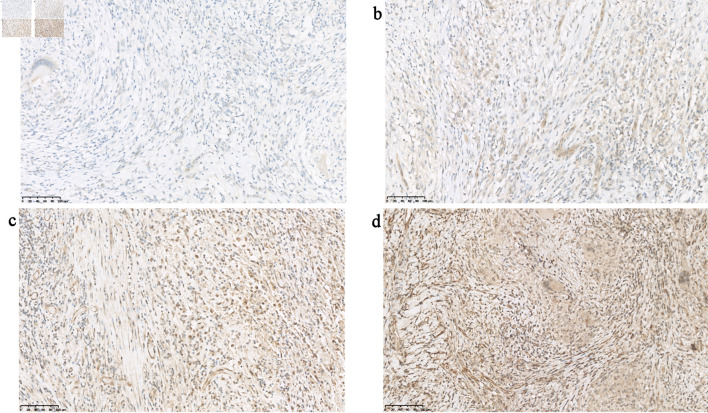
Expression of TLR4 in pericardial tissue from different patients (scale bars = 100 µm for all panels). TLR4: toll-like receptor 4.

**Figure 5:**
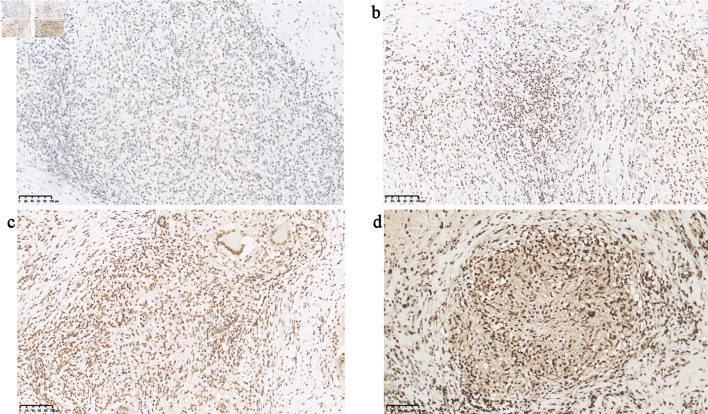
Expression of HMGB1 in pericardial tissue from different patients (scale bars = 100 µm for all panels). HMGB1: high-mobility group box 1.

Additionally, TLR4 was expressed in 21 (87.5%) specimens, and two specimens from patients with idiopathic constrictive pericarditis showed negative expression. Figure 4 showed the expression of TLR4 in different specimens. HMGB1 expression was observed in the pericardial tissue specimens of all patients, and Figure 5 presented the expression of HMGB1 in different specimens.

### Association between TLR4, HMGB1 and α-SMA

To determine the potential association between the expression of TLR4, HMGB1 and fibrosis, a correlation analysis was further performed to examine the relationship between the expressions of TLR4, HMGB1 and key fibrosis-related proteins. Since Col-III was significantly expressed in the pericardial specimens of all patients, the association between TLR4, HMGB1 and α-SMA was analyzed.

**Figure 6:**
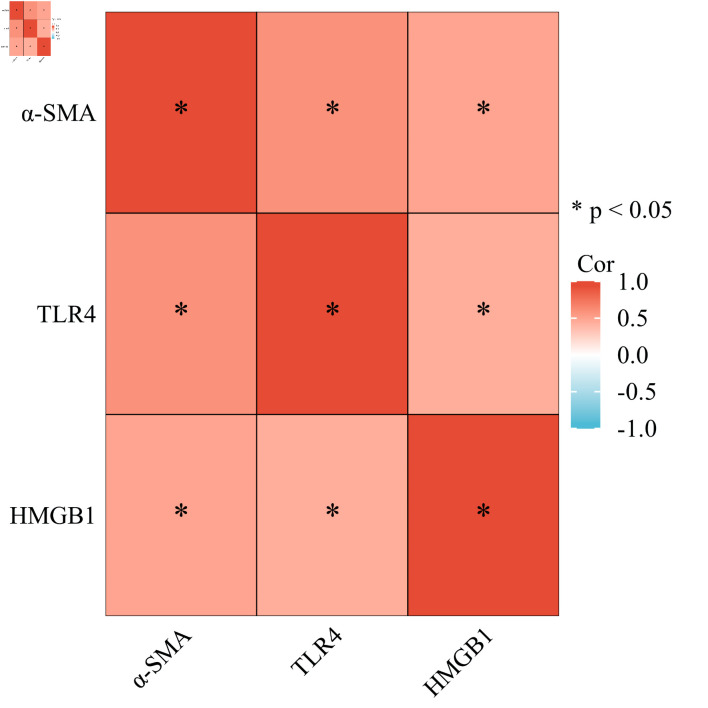
Association between TLR4, HMGB1 and α-SMA. TLR4: toll-like receptor 4; α-SMA: α-smooth muscle actin; HMGB1: high-mobility group box 1.

As shown in Figure 6, the correlation coefficient between TLR4 and α-SMA was 0.529 (P = 0.008), while the correlation coefficient between HMGB1 and α-SMA was 0.516 (P = 0.010). Additionally, a significant correlation was observed between TLR4 and HMGB1, with a correlation coefficient of 0.844 (P < 0.001).

### Association between TLR4, HMGB1 and postoperative LCOS

**Figure 7:**
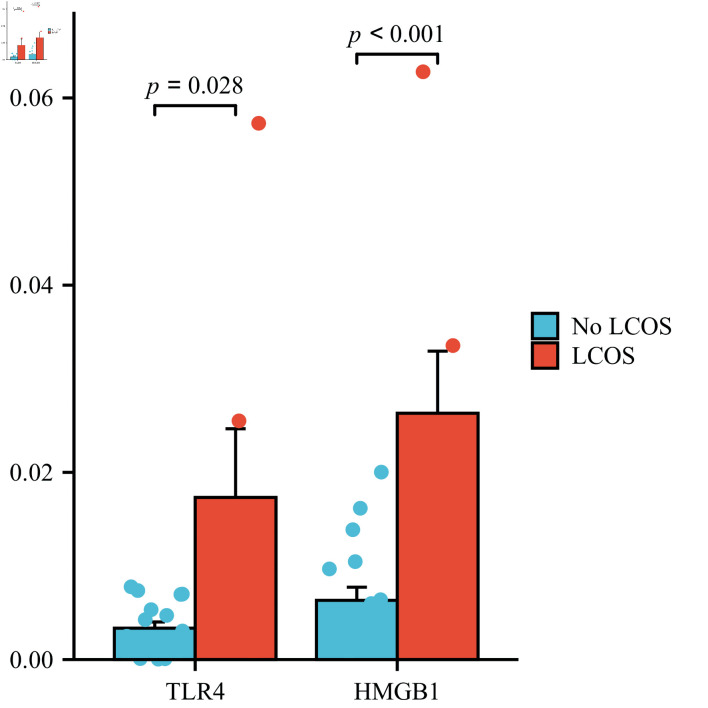
Expression of TLR4 and HMGB1 in patients with and without postoperative LCOS. LCOS: low cardiac output syndrome; TLR4: toll-like receptor 4; HMGB1: high-mobility group box 1.

**Figure 8:**
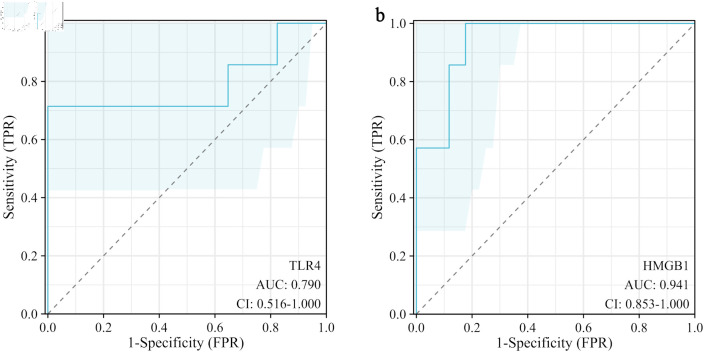
ROC curve of TLR4 (a) and HMGB1 (b) for predicting postoperative LCOS. LCOS: low cardiac output syndrome; TLR4: toll-like receptor 4; HMGB1: high-mobility group box 1; ROC: receiver operating characteristic; AUC: area under the curve; CI: confidence interval; FPR: false positive rate.

The MOD values of TLR4 and HMGB1 in pericardial tissue were significantly higher in patients who developed postoperative LCOS than in those who did not, with median values of 0.011 vs. 0.002 (P = 0.028) for TLR4 and 0.021 vs. 0.004 (P < 0.001) for HMGB1, respectively (Fig. 7). The effect sizes derived from the Mann–Whitney U test were 0.447 for TLR4 and 0.681 for HMGB1. Further analysis using ROC curves showed that the expression of TLR4 and HMGB1 had potential predictive value for the development of postoperative LCOS. The AUC of TLR4 for predicting postoperative LCOS was 0.790, with 95% confidence interval (CI) of 0.516–1.000 (Fig. 8a); the AUC of HMGB1 for predicting postoperative LCOS was 0.941, with 95% CI of 0.853–1.000 (Fig. 8b).

## Discussion

This study explored the associations of TLR4 and HMGB1 with pericardial fibrosis and the occurrence of postoperative LCOS through immunohistochemical analysis of pericardial tissues and clinical data analysis in 24 patients with constrictive pericarditis. It provided new biological evidence for understanding the disease progression of constrictive pericarditis and the mechanisms underlying postoperative adverse outcomes.

Most current studies on constrictive pericarditis have focused on clinical diagnosis and treatment, while basic research on the pathogenesis and progression of the disease remains significantly scarce. Our study confirmed via immunohistochemistry that all patients exhibited marked expression of Col-III and α-SMA in pericardial tissues, with particularly prominent Col-III expression. This indicated that the fibrotic process was widespread in the pericardial tissues of these patients. We found that both TLR4 and HMGB1 were positively correlated with α-SMA expression, which is a marker reflecting the activity of fibrosis, suggesting that TLR4 and HMGB1 may be involved in the pericardial fibrotic process in patients with constrictive pericarditis. However, there was no literature reporting which proteins expressed in the pericardial tissue affected cardiac function and postoperative outcomes in constrictive pericarditis. Based on this, we analyzed the associations between TLR4, HMGB1, and relevant clinical events. The expression levels of TLR4 and HMGB1 in pericardial tissues were significantly higher in patients who developed postoperative LCOS than in those who did not, and they showed potential predictive value for the occurrence of postoperative LCOS.

Studies have shown that the pericardium plays a unique role in cardiac development. Some cells in the pericardium may detach, invade the myocardium, and differentiate into cardiac vascular smooth muscle cells and fibroblasts, thereby playing a crucial role in maintaining myocardial integrity [17]. As the heart matures, the function of the pericardium stabilizes. However, animal model studies have suggested that myocardial injury could reactivate the pericardium, enabling it to participate in myocardium repair and the fibrotic process [18]. During acute cardiac ischemic injury, activation of the Wnt/β-catenin signaling pathway is observed in the pericardium, and inhibition of this pathway further suppresses myocardial infarction-induced fibrosis [19]. Therefore, the pericardium may play an important role in cardiac development, repair and disease progression, while the crosstalk between the pericardium and myocardium may also influence cardiac function to some extent [20].

TLR4 is a core regulator of innate immunity, and a prospective cohort study has found that TLR4 is independently associated with active tuberculosis infection and may be involved in the pathogenesis and progression of tuberculosis [21]. In our study, the expression of TLR4 was detected in almost all specimens of tuberculous pericarditis. There was a single-cell RNA sequencing study revealing that activation of TLR4 in fibroblasts was a major driver of cardiac fibrosis, confirming the direct role of TLR4 in regulating fibroblasts [22]. Additionally, as a hub molecule for inflammatory signaling, TLR4 plays an important role in pericardial-myocardial crosstalk. A study has shown that specific activation of pericardial TLR4 significantly upregulated the expression and secretion of pro-inflammatory cytokines via the NF-κB pathway, and these cytokines not only accumulate in the pericardial cavity but also diffuse to adjacent myocardium, forming a local inflammatory microenvironment [23]. Induction of myocardial inflammation could promote fibrosis, leading to deterioration of contractile function and other functional impairments [24]. In our study, the association between TLR4 expression in pericardial tissue and postoperative LCOS might also be attributed to TLR4-mediated pericardial-myocardial crosstalk under inflammatory stimulation, which induced myocardial fibrosis and thereby affected the occurrence of postoperative LCOS.

Furthermore, HMGB1 is an important endogenous ligand of TLR4. The signaling pathway initiated by their binding could drive the activation of cardiac fibroblasts and the fibrotic process, and the inhibition of HMGB1 could reduce TLR4 expression and block pro-fibrotic effects [25]. However, most studies on HMGB1 are limited to cell and animal models. We found that HMGB1 was widely expressed in pericardial specimens from constrictive pericarditis patients, with positive staining observed in the nucleus, cytoplasm and extracellular space. Moreover, HMGB1 expression was significantly positively correlated with TLR4 expression, suggesting that HMGB1 and TLR4 might synergistically participate in pericardial fibrosis and pericardial-myocardial crosstalk, thereby influencing the occurrence of postoperative LCOS.

### Limitations

Our findings suggested that TLR4 and HMGB1 might provide useful information for surgical risk stratification, allowing early identification of patients at high risk for postoperative LCOS. Prospectively, TLR4 and HMGB1 could serve as candidate biomarkers for risk assessment in clinical practice. However, this study had several limitations. First, the sample size was small, and the study was single-centered, leading to unavoidable biases. Owing to the restricted sample size, it was not feasible to conduct multivariate analysis to assess whether TLR4 and HMGB1 independently influence the occurrence of postoperative LCOS, and the analyses were exploratory in nature. Second, the etiology of constrictive pericarditis was dominated by tuberculosis, which might restrict the generalizability of our results. And the absence of a healthy pericardial control group was also a limitation. Third, while immunohistochemistry could reflect protein expression level, it could not clarify functional mechanisms. Meanwhile, regional differences in protein expression across different pericardial regions were not specifically analyzed. Additionally, this study did not obtain myocardial specimens, so it was unable to directly confirm the pericardial-myocardial crosstalk in constrictive pericarditis and the specific mechanisms, by which TLR4 and HMGB1 expression in the pericardium affected postoperative LCOS.

### Conclusions

This study suggested that TLR4 and HMGB1 might be involved in the process of pericardial fibrosis, and the expressions of TLR4 and HMGB1 were significantly associated with the occurrence of postoperative LCOS. These findings offered new insights into understanding the pathological mechanisms of constrictive pericarditis and also provided potential biomarkers for postoperative risk assessment.

## Supplementary Material

Suppl 1 Main preoperative therapeutic regimens of the study patients.

## Data Availability

The data supporting the findings of this study are available from the first or the corresponding author upon reasonable request.

## Abbreviations

LCOS: low cardiac output syndrome
TLRs: toll-like receptors
α-SMA: α-smooth muscle actin
HMGB1: high-mobility group box 1
Col-III: collagen III
H&E: hematoxylin and eosin
MOD: mean optical density
IOD: integrated optical density
