# Supplementary material for: The Role of High-Mobility Group Box 1/Toll-Like Receptor 4 in Pericardial Fibrosis and Postoperative Low Cardiac Output Syndrome in Constrictive Pericarditis
Source: Cardiol Res. 2026 Jun 5;17(3):239–47. doi: 10.14740/cr2228 (PMC13278691; doi:10.14740/cr2228)
Supplement: Suppl 1 — Main preoperative therapeutic regimens of the study patients. [file cr-17-03-239-s001.docx]

**Suppl 1.** Main preoperative therapeutic regimens of the study patients

| Patient ID | Duration of anti-TB, months | Anti-TB regimens | Diuretic | Corticosteroid | NSAIDs | Metformin | Pericardial biopsy |
| --- | --- | --- | --- | --- | --- | --- | --- |
| Patient 1 | 3 | HRELfx | Yes | No | No | No | No |
| Patient 2 | 1.5 | HRZE | Yes | No | No | No | No |
| Patient 3 | 2 | HRZELfx | Yes | No | No | No | No |
| Patient 4 | 0.2 | HELfx | Yes | No | No | No | No |
| Patient 5 | 4 | HREMfx | Yes | No | No | No | No |
| Patient 6 | 5 | HRE | Yes | No | No | No | No |
| Patient 7 | / | / | Yes | No | No | No | No |
| Patient 8 | 1.8 | HRZE | Yes | No | No | No | No |
| Patient 9 | 1.5 | HZEMfx | Yes | No | No | No | No |
| Patient 10 | 1.2 | HRZEMfx | Yes | No | No | No | No |
| Patient 11 | 0.9 | HRELfx | Yes | No | No | No | No |
| Patient 12 | 3 | HRE | Yes | No | No | No | No |
| Patient 13 | 1.2 | HRELfx | Yes | No | No | No | No |
| Patient 14 | 0.6 | HELfxLzd | Yes | No | No | No | No |
| Patient 15 | 4 | HRELfx | Yes | No | No | No | No |
| Patient 16 | 2.2 | HRZELfx | Yes | No | No | No | No |
| Patient 17 | 2 | HRZE | Yes | No | No | No | No |
| Patient 18 | 1 | HRELfx | Yes | No | No | No | No |
| Patient 19 | 1 | HEMfx | Yes | No | No | No | No |
| Patient 20 | 2 | HRZE | Yes | No | No | No | No |
| Patient 21 | 0.5 | HRZE | Yes | No | No | No | No |
| Patient 22 | 1.5 | HRZE | Yes | No | No | No | No |
| Patient 23 | 1.5 | HRZE | Yes | No | No | No | No |
| Patient 24 | / | / | Yes | No | No | No | No |

TB, tuberculosis; NSAIDs, non-steroidal anti-inflammatory drugs; H, isoniazid; R, rifampicin; E, ethambutol; Lfx, levofloxacin; Z, pyrazinamide; Mfx, moxifloxacin; Lzd, linezolid
